# The Impact of Metal-Based Nanoparticles Produced by Different Types of Underwater Welding on Marine Microalgae

**DOI:** 10.3390/toxics11020105

**Published:** 2023-01-22

**Authors:** Konstantin Pikula, Konstantin Kirichenko, Vladimir Chernousov, Sergey Parshin, Alexander Masyutin, Yulia Parshina, Anton Pogodaev, Alexander Gridasov, Aristidis Tsatsakis, Kirill Golokhvast

**Affiliations:** 1 Polytechnical Institute, Far Eastern Federal University, 10 Ajax Bay, Russky Island, Vladivostok 690922, Russia; 2 Siberian Federal Scientific Centre of Agrobiotechnology, Centralnaya Str., Presidium, Krasnoobsk 633501, Russia; 3 Peter the Great St. Petersburg Polytechnic University, 29 Polytechnicheskaya Str., St. Petersburg 195251, Russia; 4 Faculty of Biology, Lomonosov Moscow State University, 1-12 Leninskie Gory, Moscow 119991, Russia; 5 St. Petersburg University, 7–9 Universitetskaya Embankment, Str., St. Petersburg 199034, Russia; 6 Medical School, University of Crete, 13 Andrea Kalokerinou, Heraklion 71003, Greece

**Keywords:** toxicity, bioassay, underwater welding, flow cytometry, welding wire, risk assessment

## Abstract

Underwater wet welding is commonly used in joining pipelines and in underwater construction. Harmful and hazardous compounds are added to many flux-cored wires for underwater welding and cutting, and can have a negative impact on marine life. The specific objective of this study was to evaluate the aquatic toxicity of two suspension samples obtained using welding electrode and flux-cored wire in marine microalgae *Attheya ussuriensis* and *Porphyridium purpureum*. Growth rate inhibition, cell size, and biochemical changes in microalgae were evaluated by flow cytometry. The results of the bioassay demonstrated that the suspension obtained after welding with electrode had an acute toxic impact on diatomic microalgae *A. ussuriensis*, and both tested suspensions revealed chronic toxicity in this microalga with a 40% growth rate inhibition after exposure to 40–50% of prepared suspensions for 7 days. Red algae *P. purpureum* revealed tolerance to both suspensions caused by exopolysaccharide covering, which prevents the toxic impact of metal cations such as Al, Ti, Mn, Fe, and Zn, which are considered the main toxic components of underwater welding emissions.

## 1. Introduction

Welding is a complex procedure for the joining of two similar or dissimilar metals by melting the parts together with heating and subsequent cooling [1]. Welding in underwater conditions has many applications, such as offshore construction for oil and gas exploration and transportation, repair and maintenance of underwater pipelines and ships, construction of large ships beyond the capacity of existing docks, salvaging of sunk vessels, and others [2]. Underwater welding is a significantly more complex technique compared to normal welding [3], which can be classified as dry, local dry, and wet welding, which are directly performed in water without any special drainage facilities [4]. This work focused only on underwater wet welding, as it has the most extensive applications in marine engineering [5] and represents a possible threat to aquatic environments.

Many studies constantly appear to suggest new methods for the improvement in stability and quality of underwater wet welding [6,7,8]. The work of Surojo et al. (2020) reported that underwater welding related to high risks for welders, such as electric shock. Another related risk was hydrogen and oxygen, which can build potentially explosive pockets of gas. The other important risks are the difficulty of weld inspection and the possibility of low quality connection compared to the structures welded in air [9]. However, underwater wet welding still has not been considered in terms of environmental life cycle or risk assessment. The evaluation of environmental risks caused by the release of underwater welding emissions into the water should be implemented in a quality management system of the offshore and coastal welding industry.

Despite the fact that welding processes are well studied in terms of air pollution and subsequent health and environmental risks [10,11,12,13], very little was found in the literature on the question of the impact of underwater wet welding on aquatic environments. Nevertheless, underwater welding might be a significant source of water pollution due to metal-based nanoparticles. Our previous studies demonstrated that underwater welding results in the emission of metal-based particulate matter into the surrounding water [14,15].

Regular welding is reported to be a source of metal ions and metal-based particles, including known toxic elements like Cr and Cr(VI), Mn, Ni, and Al [16,17,18]. Tashiro et al. (2010) described a simulation model which theoretically clarifies the fume formation mechanism [19]. This model also revealed the formation of secondary particles of sizes up to 300 nm consisting of small particles with a size of several tens of nanometers. In underwater conditions, previous works described only the formation of metal spatter droplets in sizes of around several millimeters [20]. Xu et al. (2020) established the model of hollow spherical metal droplet formation in wet welding [21]. However, the emissions of nanoparticles during underwater welding have not been taken into consideration. Our study was one of the first assays in this direction with the attempt to evaluate possible risks of underwater welding emissions in aquatic biota.

The purpose of this investigation was to assess the possible impact of underwater welding on aquatic environments with the bioassay model of two marine microalgae species, namely *Attheya ussuriensis* and *Porphyridium purpureum*. This work provided an important opportunity to advance the understanding of possible risks related to the emissions of underwater welding.

## 2. Materials and Methods

### 2.1. Sample Preparation and Characterization

The model of underwater welding was performed in an aquarium filled with seawater. Seawater was collected from the Ajax Bay in the Peter the Great Gulf (the Sea of Japan, Far Eastern Russia) in 40-L sterile containers. The salinity of the obtained water was 33 ± 1‰. The same water then was used for microalgae bioassay.

The collected water was filtered through 0.45-μm filters and poured into a 160-L glass aquarium. Particle suspension was collected from the aquarium after 60 s of welding. The welding was performed in two different ways, as described below.

The first process was performed with an arc welding electrode Arcair Sea-Weld (Victor Technologies International, Inc., Chesterfield, MO, USA), 8.0 mm in diameter and 356 mm in length (5/6″ × 14″), cat. no.: 42-059-007. The electrode covering has three layers: iron oxide, aluminum, and plasticized vinyl. The welding was performed in the lower position at direct-current straight polarity at a current of 120 A, and welding speed 256 mm/min.

The second process was performed with a flux-cored wire PPS-APL2, 1.6 mm in diameter (Educational Scientific and Technical Center “Svarka”, St. Petersburg, Russia; Russian technical specification 1274-001-83763787). Flux composition was CaF_2_-TiO_2_-Na_3_AlF_6_-FeMn-Fe-Ni. The average welding current was 230 A, the arc voltage 38 V, and the wire feed rate 4.5 m/min.

Five millimeter thick metal plates of commercial quality carbon steel, Russian standard VSt3sp according to GOST 19903-74, were used as base material. The composition of the steel was as followed, wt.%: 0.14–0.22 C; 1.12–0.3 Si; 0.4–0.65 Mn; <0.3 Ni; <0.05 S, <0.05 P, <0.3 Cr; <0.3 Cu, <0.08 As, <100 Fe. The length of the deposited beads was 100 mm.

In further discussion, the samples obtained after using the arc welding electrode will be named ELD, and the samples obtained after using the flux-cored wire will be named WR. The obtained samples were used for toxicological study.

For the characterization, the particles emitted during the underwater welding were centrifuged and washed with distilled water twenty times, then one time with ethanol, and one more time with distilled water. For further analysis, 10 μL of the obtained particle suspension was placed on a copper mesh covered with a formvar film, and then dried at room temperature. The morphology of obtained particles was studied with analytical transmission electron microscope JEM 2100 (JEOL, Tokyo, Japan). TEM studies were carried out at the shared Research Facility “Electron Microscopy in Life Sciences” at Moscow State University.

The composition of metallic elements was measured in the suspensions of underwater welding after 7 days of suspension preparation. This analysis was performed with an ICP-MS spectrometer (Agilent 7700×, Agilent Technologies, Santa Clara, CA, USA). Sample preparation and measurement methodology of ICP-MS analysis were described in detail in our previous work [14].

### 2.2. Microalgae Cultures and Exposure

Microalgal cultures were provided by The Resource Collection *Marine biobank* of the National Scientific Center of Marine Biology, Far Eastern Branch of the Russian Academy of Sciences (NSCMB FEB RAS). The toxicity bioassay of underwater welding suspension was carried out on two marine microalgae isolated from the Ajax Bay in the Peter the Great Gulf (the Sea of Japan, Far Eastern Russia), namely the diatom species *Attheya ussuriensis* Stonik, Orlova et Crawford, 2006 (Bacillariophyta) and a red algae *Porphyridium purpureum* (Bory de Saint-Vincent) Drew et Ross, 1965 (Rhodophyta). The microalgae model is a sensitive bioindicator of aquatic pollution [22,23]. At the same time, microalgae is a crucial element of all aquatic trophic chains and the main producer of organic matter in the aquatic environment [24]. The particular microalgae species were selected based on their abundance among microalgae in the Sea of Japan [25] and their suitability as test organisms in ecotoxicology [26,27,28].

Culturing of the microalgae and toxicity test conditions were maintained in accordance with the guidance of OECD No.201 [29] with minor modifications as previously described [30,31]. Microalgae were cultured with Guillard’s f/2 medium [32]. Filtered (pore diameter of the filter was 0.22 μm) and sterilized seawater with salinity 33 ± 1‰, pH 8.0 ± 0.2 was used for the experiments. The cultivation was carried out at a temperature of 20 ± 2 °C with an illumination intensity of 300 μmol photons/m^2^s, and a light:dark cycle of 12:12 h. Algal cultures in the exponential growth phase were used for bioassays.

Before the experiment, microalgae cells were cultivated in 250 mL Erlenmeyer flasks. For the experiment, microalgae cells were transferred to 24-well plates where each well contained 1 mL of microalgae aliquots and 1 mL of the tested sample. The initial cell density in each well was 1.2–1.5 × 10^4^ cells/mL for both microalgae species. The wells of a control group had only microalgae aliquots with addition of 1 mL of f/2 medium. The other wells had different concentrations of welding suspensions ELD and WR, namely 2.5, 5, 10, 20, 30, 40, and 50% of the stock particle suspension from the total used volume of water (2 mL). Each concentration and control group were carried out in triplicate.

### 2.3. Flow Cytometry Analysis

Microalgae cell counting and registration of morphological and biochemical changes were carried out using flow cytometer CytoFLEX (Beckman Coulter, Indianapolis, IN, USA) with the software package CytExpert v.2.0. The changes in microalgae cells after the exposure to welding suspensions were evaluated using specific fluorescent dyes. All the measurements with each fluorescent dye were performed separately after 96 h and 7 days of exposure. Each sample was measured at a flow rate of 100 μL/min for 30 s. The emission channels were selected according to the data provided by the manufacturer (Molecular Probes, Eugene, OR, USA). The blue laser (488 nm) of the CytoFLEX flow cytometer was chosen as a source of excitation light. The data of flow cytometry measurements were expressed as mean fluorescence intensity (MFI). The endpoints of toxicity used in this work and the parameters of their registration are listed in Table 1.

The number of alive microalgae cells in each measurement was determined using FSC/SSC dot cytogram (forward scattering to side scattering ratio) which allowed for the separation of the population of events with the sizes similar to the expected size of microalgae cells. Then, nonalgal events were excluded from the separated population by the absence of chlorophyll *a* fluorescence in the emission filter FL3 (690 nm), where all the algal cells had high MFI. Dead cells were excluded from the counting by them staining with propidium iodide (PI) according to the standard bioassay protocol [33], where dead cells obtained high MFI in the emission filter FL1 (610 nm).

The level of reactive oxygen species (ROS) generation in microalgae cells was assessed using non-fluorescent dye 2′,7′-dichlorodihydrofluorescein diacetate (H_2_DCFDA), which activates in the presence of ROS [34]. The membrane potential of microalgae cells was assessed by a lipophilic, positively charged fluorescent dye 3,3′-dihexyloxacarbocyanine iodide (DiOC_6_), which is capable of binding to membranes (mitochondria and endoplasmic reticulum) and other hydrophobic negatively charged cell structures [35]. Both ROS generation and membrane potential were registered as MFI in the emission filter FL2 (525 nm) compared to the MFI of the control group in the same emission channel. The evaluation of ROS generation and membrane potential were performed separately to exclude overlapping of the emissions after staining with H_2_DCFDA and DiOC_6_. The optimal concentration of the dyes and duration of the staining for each microalgae species were chosen based on previous works [31].

To determine the size of microalgae cells, a size calibration kit F13838 (Molecular probes, USA) with the certified size distribution of 1, 2, 4, 6, 10, and 15 μm was used for the FSC emission channel.

### 2.4. Microscopic Observation

Morphological changes in microalgae cells exposed to the welding suspension were captured by optical microscope Axio Observer A1 (Carl Zeiss, Berlin, Germany) at magnification 1000×.

### 2.5. Statistical Analysis

Statistical analyses were performed by GraphPad Prism 8.0.2 (GraphPad Software, San Diego, CA, USA). The statistical significance was tested by one-way ANOVA. Normality was checked using the Shapiro–Wilk test. A value of *p* ≤ 0.05 was considered statistically significant.

## 3. Results

### 3.1. Sample Characterization

**Figure 1:**
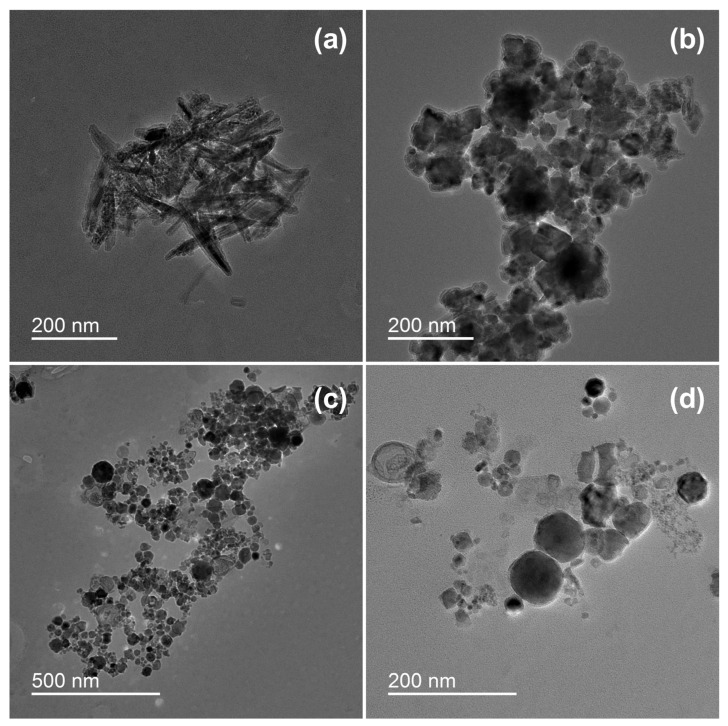
Morphology of underwater welding particles: (**a**) Spike-shaped particles of sample ELD; (**b**) Spherical particles of sample ELD; (**c**) Spherical particles of sample WR; (**d**) Spherical particles of sample WR in higher magnification. ELD, suspension obtained after using an arc welding electrode; WR, suspension obtained after using a flux-cored wire. Scale bar = 200 nm.

Transmission electron microscopy analysis revealed different structures of the particles formed by underwater welding (Figure 1). It should be highlighted that sample ELD had both spike-shaped particles (Figure 1a) and spherical particles (Figure 1b), but sample WR had only the particles in spherical form (Figure 1c,d). The analysis also revealed that spherical particles of sample ELD (Figure 1b) were agglomerated into relatively big clusters of several micrometers with strong bindings. On the other hand, the spherical particles of sample WR (Figure 1c) had loose bindings and could be easily dispersed under the dynamic water flow (Figure 1d).

The results of ICP-MS analysis demonstrated that welding suspensions increase concentrations of metallic elements in water (Table 2). Among the two samples, WR had higher concentrations of B and Si.

### 3.2. Toxicity Bioassay

**Figure 2:**
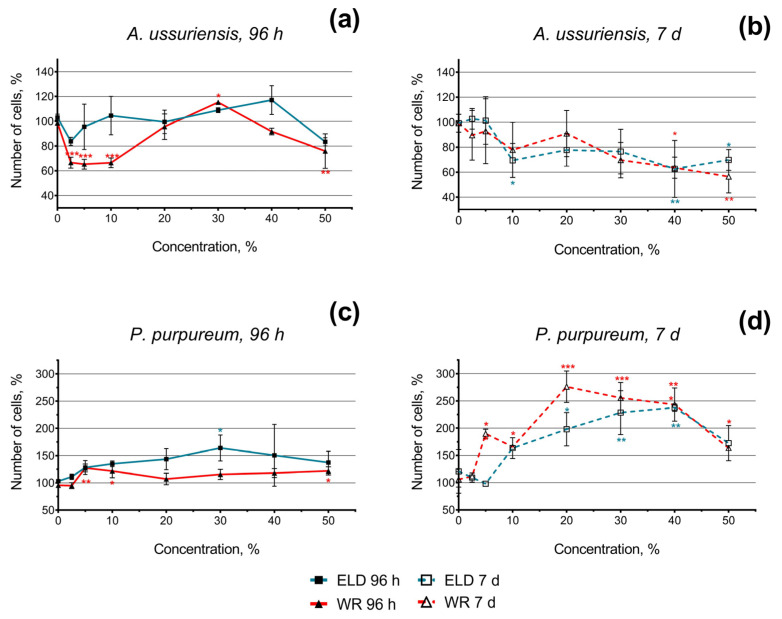
Microalgae growth rate change after 96 h and 7 days of exposure to underwater welding suspensions in seawater: (**a**) Effect of underwater welding on the growth of microalgae *A. ussuriensis* after 96 h of exposure; (**b**) Effect of underwater welding on the growth of microalgae *A. ussuriensis* after 7 days of exposure; (**c**) Effect of underwater welding on the growth of microalgae *P. purpureum* after 96 h of exposure; (**d**) Effect of underwater welding on the growth of microalgae *P. purpureum* after 7 days of exposure. ELD, suspension obtained after using an arc welding electrode; WR, suspension obtained after using a flux-cored wire. Error bars represent standard deviation. *, *p* < 0.05; **, *p* < 0.01; ***, *p* < 0.001.

The changes in the growth rate of the microalgae after 96 h and 7 days of exposure to welding suspensions are represented in Figure 2. The number of cells in the control group was counted as 100%.

In general, the diatom species *A. ussuriensis* revealed higher sensitivity to both samples compared to the red algae species *P. purpureum*. Moreover, the growth rate inhibition of *A. ussuriensis* increased over time (the effect after 7 days was higher than after 96 h). Interestingly, significant (*p* < 0.001) growth rate inhibition was observed at lower concentrations (2.5–10%) in sample WR (Figure 2a); this effect was not consistent after 7 days of exposure (Figure 2b). This observation can be a sign of acute toxicity in sample WR in diatom species *A. ussuriensis*. After chronic exposure (7 days) of *A. ussuriensis*, both underwater welding samples significantly inhibited microalgae growth rate (up to 40%) at concentrations 40 and 50% (Figure 2b).

Red algae *P. purpureum*, after 96 h of exposure, responded with moderate growth rate stimulation or demonstrated no significant change in microalgae growth (Figure 2c). This effect was significantly higher after 7 days of exposure (Figure 2d). After 7 days of exposure at the concentration of 40% and 50%, the stimulation of *P. purpureum* growth rate dramatically decreased compared to lower concentrations (Figure 2b). This observation most likely means that higher concentrations of underwater welding particles would represent a threat to *P. purpureum* at chronic exposure.

**Figure 3:**
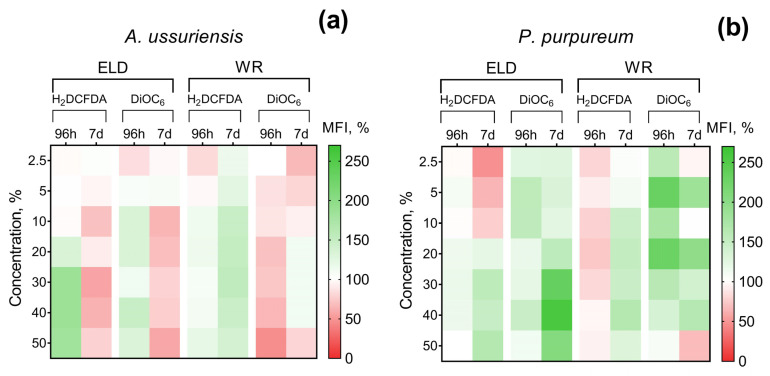
Effect of underwater welding on ROS generation and membrane potential of microalgae after 96 h and 7 days of exposure: (**a**) Changes observed for microalgae *A. ussuriensis*; (**b**) Changes observed for microalgae *P. purpureum*. ELD, suspension obtained after using an arc welding electrode; WR, suspension obtained after using a flux-cored wire; H_2_DCFDA, 2′,7′-dichlorodihydrofluorescein diacetate (ROS generation indicator); DiOC_6_, 3,3-dihexyloxacarbocyanine iodide (membrane potential indicator). All the white sections represent nonsignificant results (*p* > 0.05).

The changes in ROS generation and membrane polarization of microalgae after 96 h and 7 days of exposure to welding suspensions are represented in Figure 3. The registered MFI of H_2_DCFDA and DiOC_6_ in the control group of microalgae was counted as 100%. Therefore, increases and decreases in H_2_DCFDA fluorescence intensity can be interpreted as increases and decreases in ROS generation, respectively. An increase in DiOC_6_ fluorescence intensity means microalgae membrane hyperpolarization and a decrease in DiOC_6_ fluorescence intensity can be interpreted as membrane depolarization.

It should be noted that *A. ussuriensis*, the more sensitive species to welding suspensions (according to Figure 2), responded mostly with MFI inhibition in both fluorescent dyes (Figure 3a). At the same time, *P. purpureum* did not reveal growth rate inhibition (Figure 2c,d) and mostly responded with an increase in MFI in both fluorescent dyes (Figure 3b). The obtained results revealed that microalgae species respond with an increase in ROS generation and membrane hyperpolarization as a part of adaptation mechanisms under concentrations with lower toxicity, which corresponds with the stimulation of microalgal growth rate (Figure 2). This effect is replaced by subsequent inhibition of both parameters in cases when toxic exposure becomes critical, which correlates with the inhibition of microalgal growth rate (Figure 2). Interestingly, sample WR caused membrane depolarization of *P. purpureum* only at the highest concentration after 7 days of exposure (Figure 3b), and at this concentration, the curve of *P. purpureum* growth rate turned direction, from stimulation to inhibition.

**Figure 4:**
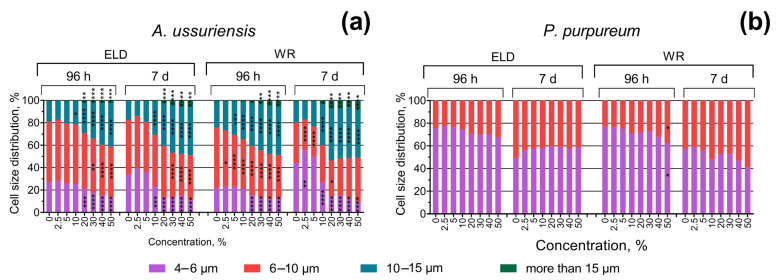
Effect of underwater welding on the size of microalgae cells after 96 h and 7 days of exposure: (**a**) Changes in cell size distribution of microalgae *A. ussuriensis*; (**b**) Changes in cell size distribution of microalgae *P. purpureum*. ELD, suspension obtained after using an arc welding electrode; WR, suspension obtained after using a flux-cored wire. *, *p* < 0.05; **, *p* < 0.01; ***, *p* < 0.001; ****, *p* < 0.0001.

The changes in the size of microalgae cells after 96 h and 7 days of exposure are represented in Figure 4. In general, the results demonstrate significant concentration- and time-dependent increases in the size of the cells of *A. ussuriensis*. *P. purpureum* demonstrated significant but not dramatic increases in cell size only after 96 h of exposure to sample WR at the highest used concentration, and this effect was not registered after 7 days of exposure (Figure 4b).

**Figure 5:**
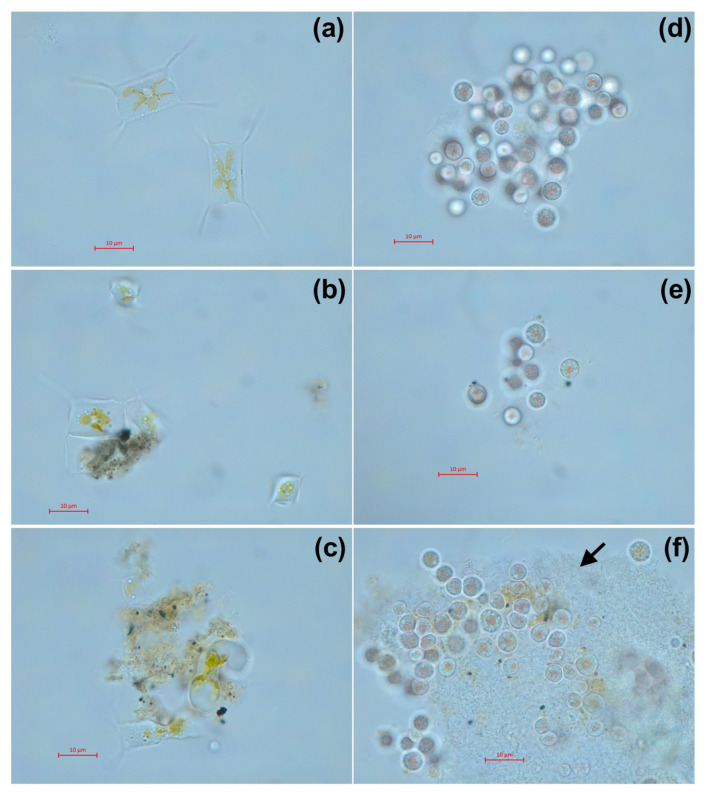
Microscopic pictures of microalgae after 7 days of exposure to welding suspensions at concentration 50%: (**a**) Control group of *A. ussuriensis* without the exposure; (**b**) *A. ussuriensis* exposed to sample ELD; (**c**) *A. ussuriensis* exposed to sample WR; (**d**) Control group of *P. purpureum* without the exposure; (**e**) *P. purpureum* exposed to sample ELD; (**f**) *P. purpureum* exposed to sample WR. ELD, suspension obtained after using an arc welding electrode; WR, suspension obtained after using a flux-cored wire. Black arrow indicates the exopolysaccharide coverage, excreted by *P. purpureum* cells. Scale bar = 10 µm.

The morphology of microalgae cells after 7 days of exposure at the highest used concentration is presented in Figure 5. Both samples of welding suspensions tend to be absorbed by microalgae cells. In case of *A. ussuriensis*, sample WR caused high deformation of the algal cells (Figure 5c). For *P. purpureum*, the exposure to WR caused an excretion of mucous to protect the cells from the toxic influence of the welding particles (Figure 5f).

## 4. Discussion

As mentioned in the introduction, the impact of underwater welding emissions on aquatic biota still have not been established, and current work suggests this question for discussion.

The obtained results demonstrated that sample WR after 60 s of welding significantly increased the concentration of Si, B, and Na in water (Table 2) compared to the sample ELD. Despite that, the difference between the toxic levels of the two welding suspensions in microalgae was not so pronounced (Figure 2), which correlates with the fact that the concentrations of elemental metals such as Al, Ti, Mn, Fe, and Zn were similar for both samples (Table 2). According to the classification of metals based on Lewis acidity [36], Al and Fe(III) are Class A hard metals, Ti is a Class B soft metal, and Mn, Fe(II), and Zn are borderline intermediate metals. The high aquatic toxicity was previously reported in Zn ions [37]. The other mentioned metals have relatively low toxicity in aquatic species; however, their mixtures with each other and with other pollutants can cause significant synergistic action [38,39,40]. Therefore, we can conclude that the chemical composition of the emitted metals have a critical importance in aquatic toxicity of underwater welding suspensions. This conclusion is in accord with multiple previous bioassays of metal-based nanoparticles in aquatic species [41,42].

Nevertheless, microalgae species *A. ussuriensis* apparently demonstrated higher sensitivity to welding suspensions because of its benthic position in the water column, which probably facilitated the contact between microalgae cells and sedimented particulate matter. As it can be expected, further sedimentation of the sample suspensions increased the toxic impact on *A. ussuriensis* with time (Figure 2b). The agglomeration of the particles of both underwater welding suspensions on the cells of *A. ussuriensis* can be seen in Figure 5b,c. Interestingly, the sample WR caused severe deformation of the cells (Figure 5c), which correlates with high membrane depolarization in this microalgae species under the exposure to WR starting from 96 h of exposure (Figure 3a). Previous studies demonstrated that diatom microalgae can be used as an effective tool for the remediation of pollutants, including metal ions, because of their high absorption potential [43]. Moreover, the deformation of diatomic cells were reported after exposure to zinc and iron [44,45,46], which correlates with our results of flow cytometry measurement and microscopic observation. The impact of metal ions results in valve or teratological deformation in diatom frustule and puts the cells under metabolic stress by increasing the lipid bioaccumulation [43]. The prolonged exposure and gradual increase in oxidative stress explain the higher chronic toxicity of underwater welding towards *A. ussuriensis*.

The opposite situation was observed for microalgae species *P. purpureum*, which is usually equally distributed in the water column [47] compared to benthic *A. ussuriensis* [48]. In the case of this microalgae species, both suspension samples stimulated the growth rate of the cells (Figure 2b), which means that toxic concentration of suspended particles and metal ions was not reached for *P. purpureum*. The stimulation of microalgal growth rate might be a demonstration of the hormesis effect [49], and further increases in concentration or duration of exposure can cause toxic impact. This assumption is in accord with the decline of the growth rate curve in *P. purpureum* with the increase in underwater suspension concentration higher than 40% (Figure 2d). The reason for the observed tolerance to metal particulates and metal ions in this species can be explained by exopolysaccharide coverage of *P. purpureum*, which protects cells from hydrophobic particulate matter [50]. Our previous study demonstrated the high tolerance of *P. purpureum* to silica [26] and ZnS nanoparticles [31], which correlates with the results of the present study. The absorption of particulate matter of sample WR by the exopolysaccharide coverage of *P. purpureum* can be seen in Figure 5f.

The changes in ROS generation and membrane polarization of microalgae cells also correlate with the hormesis effect and were increased below the toxic concentrations and inhibited when negative impacts became critical for microalgae cells (Figure 3). Compared to each other, the two tested underwater suspension samples did not reveal substantial differences in their effects on the antioxidative systems and membrane functionality of microalgae. These results also demonstrated that the chemical composition of underwater wet welding emissions was the important factor in toxicity, and similar concentrations of elemental metals in the tested samples resulted in similar effects on the systems of microalgae.

Our study demonstrated that the suspensions obtained during the two different underwater wet welding processes, namely performed with arc welding electrode and flux-cored wire, had different toxic effect on the two used microalgae species, but had little difference in comparison to each other. As the most important factor which determined the toxicity of the obtained samples, we should emphasize the concentration of elemental metals in the emissions of underwater welding and the bioavailability of these components to aquatic species.

In conclusion, it should be highlighted that underwater wet welding could possibly represent a threat to aquatic life. However, considering the massive lack of studies and the absence of any emission controls during underwater wet welding processes, there is a need for the development of standard procedures, including the evaluation of real-life emissions of underwater welding, methodology of sample collection, parameters and guidelines for sample characterizations and toxicity testing, and development of regulatory recommendations and standards, if it is required. Therefore, further studies need to support safe welding processes not only for welders and employees but also for aquatic environment.

## Figures and Tables

**Table 1:** Toxicity assessment criteria and conditions of their registration.

Endpoint	Biomarker or Parameter	CytoFLEX Emission Channel, nm
Growth rate inhibition	PI	FL1, 610
ROS generation	H_2_DCFDA	FL2, 525
Membrane potential	DiOC_6_	FL2, 525
Size	Forward scatter intensity	FSC

ROS, Reactive oxygen species; PI, Propidium iodide; FDA, Fluorescein diacetate; H_2_DCFDA, 2′,7′-dichlorodihydrofluorescein diacetate; DiOC_6_, 3,3′-dihexyloxacarbocyanine iodide.

**Table 2:** Metal content of welding suspensions in seawater registered by ICP-MS analysis.

Chemical Element	Concentration in Suspension, µg/L		
	Control	ELD	WR
^7^Li	140	144	150
^11^B	4849	4815	5944
^23^Na	939,8281	9,020,554	9,773,710
^27^Al	92	487	492
^28^Si	16,578	≤16,000	28,697
^47^Ti	2	778	788
^55^Mn	7	291	294
^56^Fe	99	5671	5813
^66^Zn	33	538	519

This table represent only the values which significantly differ compared to control. All the results of ICP-MS analysis are represented in Table S1.

## Data Availability

Not applicable.

## Supplementary Materials

The following supporting information can be downloaded at: https://www.mdpi.com/article/10.3390/toxics11020105/s1, Table S1: Results of ICP-MS analysis of two welding suspensions in seawater.
