# Supplementary material for: The Impact of Metal-Based Nanoparticles Produced by Different Types of Underwater Welding on Marine Microalgae
Source: Toxics. 2023 Jan 22;11(2):105. doi: 10.3390/toxics11020105 (PMC9966890; doi:10.3390/toxics11020105)
Supplement: Supplementary file 1 [file toxics-11-00105-s001.zip › toxics-2117236-supplementary.pdf]

toxics

MDPI

Supplementary Materials: The Impact of Metal-Based Nanoparticles Produced by Different Types of Underwater Welding on Marine Microalgae

Pukula, Konstantin Kirichenko, Vladimir Chernousov, Sergey Parshin, Alexander Masyutin, Yulia Parshina, Anton Pogodaev, Alexander Gridasov, Aristidis Tsatsakis and Kirill Golokhvast

Table S1. Results of ICP-MS analysis of two welding suspensions in seawater.

Chemical Species	Concentration in Suspension, μg/L		
	Control	ELD	WR
^{7}Li	140	144	150
^{9}Be	0	≤ 0,057	≤ 0,057
^{11}B	4849	4815	5944
^{23}Na	9398281	9020554	9773710
^{24}Mg	1166314	1119792	1181197
^{27}Al	92	487	492
^{28}Si	16578	≤ 16000	28697
^{31}P	123	86	145
^{34}S	562796	533411	559774
^{39}K	359836	346340	368331
^{44}Ca	457236	434272	454397
^{45}Sc	0	0	0
^{47}Ti	2	778	788
^{51}V	2	4	5
^{52}Cr	2	5	6
^{55}Mn	7	291	294
^{56}Fe	99	5671	5813
^{59}Co	1	1	1
^{60}Ni	6	8	9
^{63}Cu	59	71	67
^{66}Zn	33	538	519
^{71}Ga	0	0	0
^{72}Ge	0	0	1
^{75}As	2	2	2
^{78}Se	1	1	0

^{85}Rb	97	101	95
^{88}Sr	7304	7418	7141
^{89}Y	0	0	0
^{90}Zr	0	6	5
^{93}Nb	0	3	3
^{98}Mo	11	12	11
^{107}Ag	0	0	0
^{114}Cd	0	2	2
^{118}Sn	1	5	4
^{121}Sb	1	1	1
^{125}Te	≤ 0,19	≤ 0,19	0
^{133}Cs	0	0	0
^{137}Ba	9	17	16
^{139}La	0	0	0
^{140}Ce	0	0	0
^{141}Pr	0	0	0
^{146}Nd	0	0	0
^{147}Sm	0	0	0
^{151}Eu	0	0	0
^{157}Gd	0	0	0
^{159}Tb	0	0	0
^{163}Dy	0	0	0
^{165}Ho	0	0	0
^{166}Er	0	0	0
^{169}Tm	≤ 0,00044	0	0
^{172}Yb	≤ 0,0022	0	0
^{175}Lu	0	0	0
^{178}Hf	0	0	0
^{181}Ta	≤ 0,027	0	0
^{184}W	0	1	1
^{187}Re	0	0	0
^{205}Tl	0	0	0
^{208}Pb	1	4	3
^{209}Bi	0	0	0
^{232}Th	0	0	0

^{238}U	3	3	3
